# Liuwei Dihuang pills attenuates cognitive dysfunction in *APP/PS1* mice through restoring mitochondrial homeostasis and activating the IRS/AKT/GSK3β insulin signaling pathway

**DOI:** 10.3389/fphar.2026.1873606

**Published:** 2026-08-20

**Authors:** Junying Song, Yong Yuan, Mao Wang, Junlin Li, Rui Ding, Yaquan Jia, Zichuang Wang, Zhishen Xie, Zhonghua Li, Zhenqiang Zhang

**Affiliations:** 1 Academy of Chinese Medical Sciences, Henan University of Chinese Medicine, Zhengzhou, China; 2 Collaborative Innovation Center of Research and Development on the Whole Industry Chain of Yu-Yao, Zhengzhou, China; 3 Collaborative Innovation Center of Prevention and Treatment of Major Diseases By Chinese and Western Medicine, Zhengzhou, China; 4 The Second Clinical Medical College of Henan University of Chinese Medicine, Zhengzhou, China

**Keywords:** alzheimer’s disease (AD), cognitive deficits, IRS/AKT/GSK3β insulin signaling pathway, Liuwei Dihuang Pills (LWDHP), mitochondrial homeostasis

## Abstract

**Introduction:**

Alzheimer’s disease (AD) features progressive cognitive decline, Aβ aggregation, neurofibrillary tangles, mitochondrial dysfunction and central insulin resistance, lacking effective disease-modifying treatments. Liuwei Dihuang Pills (LWDHP) exert neuroprotective effects, while its dual regulatory mechanisms targeting mitochondrial homeostasis and cerebral IRS/AKT/GSK3β insulin signaling remain unclear.

**Methods:**

*APP/PS1* transgenic mice were used as AD models. Morris water maze assessed spatial cognition; immunofluorescence detected Aβ and MAP2; transmission electron microscopy observed neuronal/synaptic ultrastructure; western blot quantified synaptic proteins and insulin signaling cascade molecules.

**Results:**

LWDHP significantly rescued spatial learning and memory deficits, reduced cerebral Aβ plaques and tau hyperphosphorylation. LWDHP balanced mitochondrial fusion-fission via upregulating PGC-1α/MFN2 and downregulating FIS1, elevated synaptic structural proteins (MAP2, PSD-95, SYN), restored cerebral insulin sensitivity by elevating InsR, normalizing IRS-1/AKT/GSK3β phosphorylation, and suppressing tau hyperphosphorylation.

**Discussion:**

LWDHP alleviates AD cognitive impairment through dual regulation of mitochondrial homeostasis and IRS/AKT/GSK3β insulin signaling, providing solid preclinical evidence for its anti-AD application.

## 1 Introduction

Alzheimer’s disease (AD) is an age-related, progressive neurodegenerative disorder characterized by irreversible memory loss and progressive cognitive decline, accounting for approximately 60%-80% of all dementia cases (Yepes, 2021). Its core pathological hallmarks are the extracellular aggregation of amyloid β (Aβ) peptides into senile plaques (SPs) and the intracellular formation of neurofibrillary tangles (NFTs) composed of hyperphosphorylated tau protein-both of which are most prominent in the hippocampus and cerebral cortex, brain regions critical for learning and memory (Yepes, 2021; Nasreddine et al., 2023; Basaly et al., 2021). These pathological changes are accompanied by a cascade of detrimental events, including extensive neuronal loss, brain hypometabolism, synaptic dysfunction, mitochondrial impairment, and persistent neuroinflammation, which collectively drive the progressive deterioration of cognitive function (Nasreddine et al., 2023; Basaly et al., 2021). Emerging research has refined the understanding of AD pathogenesis by positioning metabolic dysregulation and mitochondrial impairment, as core upstream triggers preceding classic Aβ and tau lesions, rather than secondary pathological outcomes induced by plaque deposition (Lanzillotta et al., 2024; D'Alessandro et al., 2025). Mitochondrial structural and functional decline initiates excessive reactive oxygen species burst, neuronal energy depletion and subsequent synaptic damage, which substantially accelerates Aβ overproduction, abnormal tau hyperphosphorylation and eventually irreversible neuronal degeneration (Shoshan-Barmatz et al., 2018; Ashleigh et al., 2023). Despite decades of intensive research and recent advances in targeted therapies (e.g., FDA-approved anti-Aβ monoclonal antibodies), the complex multifactorial mechanisms underlying AD onset and progression remain incompletely understood, and effective long-term therapeutic strategies to halt or reverse disease progression are still lacking.

In recent years, a growing body of evidence has reshaped our understanding of AD pathogenesis by linking it to metabolic dysfunction, particularly brain insulin resistance. This has led to the conceptualization of AD as “type 3 diabetes”-a term highlighting the shared features of insulin signaling impairment, glucose metabolic disorder, and neurodegeneration between AD and type 2 diabetes mellitus (T2D) (González et al., 2022; Kciuk et al., 2024). Brain insulin plays a pivotal role beyond glucose regulation: it exerts neuroprotective and trophic effects by modulating neuronal growth, energy metabolism, mitochondrial function, synaptic plasticity, and cognitive processes (Tyagi and Pugazhenthi, 2021). The insulin signaling pathway in the brain, primarily localized in the cortex and hippocampus (Joshi et al., 2017), relies on key downstream molecules including insulin receptor substrate (IRS), phosphatidylinositol 3-kinase (PI3K), protein kinase B (AKT), and glycogen synthase kinase-3β (GSK-3β). Dysregulation of this pathway-manifested by reduced insulin receptor (InsR) expression, impaired IRS phosphorylation, and aberrant activation of GSK-3β, which has been consistently observed in the brains of AD patients and transgenic AD models (Dewanjee et al., 2022; Tyagi and Pugazhenthi, 2021). For instance, postmortem studies have documented reduced InsR levels in the hippocampus and cortex of AD patients, while animal models (e.g., *APP/PS1* mice) exhibit early impairments in IRS/AKT/GSK3β signaling prior to the onset of overt Aβ or tau pathology (Kciuk et al., 2024; Dewanjee et al., 2022; Tyagi and Pugazhenthi, 2021). This insulin resistance not only disrupts synaptic plasticity and memory formation directly but also exacerbates AD-specific pathology: activated GSK-3β promotes tau hyperphosphorylation and NFT formation, while impaired insulin signaling reduces Aβ clearance and enhances its aggregation (Abdalla, 2024). Mitochondrial dysfunction has emerged as another early and central driver of AD pathogenesis, occurring even before the accumulation of Aβ plaques and NFTs-suggesting it may be a “primary risk factor” rather than a secondary consequence of disease (Lanzillotta et al., 2024). Mitochondria are critical for neuronal survival, as they supply over 90% of the energy required for synaptic transmission and maintain redox homeostasis by regulating reactive oxygen species (ROS) production. In AD, mitochondria exhibit a spectrum of defects: ultrastructural damage (e.g., swollen cristae, fragmentation), reduced oxidative phosphorylation efficiency, dysregulated fusion-fission dynamics, and impaired mitophagy (the process of clearing damaged mitochondria) (D'Alessandro et al., 2025; Shoshan-Barmatz et al., 2018; Ashleigh et al., 2023). These impairments not only trigger energy deficits that disrupt synaptic function but also increase ROS production, leading to oxidative damage to lipids, proteins, and DNA-further amplifying neuronal injury and cognitive decline. Notably, mitochondrial function and insulin signaling are tightly interconnected, forming a bidirectional regulatory loop that is disrupted in AD. On one hand, insulin signaling modulates mitochondrial metabolism: activated AKT promotes the expression of peroxisome proliferator-activated receptor gamma coactivator 1 alpha (PGC-1α), a master regulator of mitochondrial biogenesis and oxidative metabolism (Pradeepkiran and Reddy, 2020). Reduced PGC-1α expression in AD leads to decreased mitochondrial mass and function, while its preservation has been shown to inhibit Aβ production and ameliorate neuronal loss (Sweeney and Song, 2016). On the other hand, mitochondrial dysfunction impairs insulin sensitivity: damaged mitochondria generate excessive ROS, which oxidizes IRS proteins and inhibits insulin signal transduction. Key regulators of mitochondrial dynamics also contribute to this cross-talk: mitofusin 2 (MFN2), a protein governing mitochondrial fusion, protects against tau toxicity by maintaining mitochondrial integrity-with Mfn2 overexpression reversing mitochondrial fragmentation, tau pathology, and cognitive deficits in aged tauopathy mice (Wang et al., 2025). Conversely, excessive mitochondrial fission, driven by the interaction between dynamin-related protein 1 (Drp1) and fission 1 (FIS1), exacerbates Aβ-mediated neuropathology and cognitive decline in AD models (Zhu et al., 2025). Disrupted mitophagy further compounds these issues: impaired clearance of damaged mitochondria increases oxidative stress and energy deficits, which in turn promote Aβ and tau accumulation and disrupt synaptic plasticity. Despite the recognition that insulin resistance and mitochondrial dysfunction are core contributors to AD, the molecular mechanisms linking these two pathways remain incompletely defined. This knowledge gap has hindered the development of therapies targeting both processes simultaneously.

Liuwei Dihuang Pills (LWDHP), a classic Chinese herbal formula with a history of over 1,000 years in treating age-related disorders, has recently gained attention for its potential neuroprotective effects (Song et al., 2022a). Composed of six botanical drugs (*Rehmannia glutinosa, Cornus officinalis, Dioscorea opposita, Alisma orientale, Poria cocos, and Paeonia suffruticosa*). In recent years, a series of studies from our group have demonstrated that Liuwei Dihuang Pills (LWDHP) exert anti-Alzheimer’s disease (AD) effects through coordinated modulation of multiple signaling axes-including the RAGE/LRP1 receptor system, RAGE-dependent MMP-2/MMP-9 activation, and the PI3K/Akt/FoxO3a pathway-thereby conferring dual protection to both cerebral microvasculature and neurons (Song et al., 2022a; Yuan et al., 2023; Jia et al., 2022; Song et al., 2022b; Ding et al., 2024). Concurrently, accumulating evidence implicates mitochondrial dysfunction and central insulin resistance as early and pathogenically significant contributors to AD progression. Nevertheless, whether and how LWDHP ameliorates AD-associated cognitive deficits via concurrent restoration of brain insulin sensitivity and mitochondrial homeostasis remains unexplored.

This study aimed to explore whether LWDHP alleviates AD-related cognitive deficits in *APP/PS1* transgenic mice by regulating cerebralimpaired insulin signaling pathway and mitochondrial homeostasis. We further systematically elucidated the molecular mechanisms responsible for its neuroprotective actions, focusing on the preservation of synaptic integrity, restoration of mitochondrial dynamics, and reactivation of the IRS-1/Akt/GSK-3β insulin signaling pathway. Collectively, our findings provide solid preclinical evidence for the translational potential of LWDHP in the prevention and treatment of AD, and lay a mechanistic and experimental basis for its future clinical application.

## 2 Materials and methods

### 2.1 Materials

Liuwei Dihuang Pills (LWDHP; concentrated tablet formulation; batch no.: 18,110,401; specification: 1.44 g per 8 tablets; National Drug Approval No.: Z41022128) were procured from Zhongjing Wanxi Pharmaceutical Co., Ltd. (Nanyang, Henan Province, China). LWDHP is a standardized compound herbal preparation listed in the *Chinese Pharmacopoeia (2015 edition)*. It is manufactured under Good Manufacturing Practice (GMP), compliant conditions using six authenticated botanical ingredients (*Rehmannia glutinosa Libosch.*, *Cornus officinalis Siebold & Zucc.*, *Paeonia suffruticosa Andrews*, *Dioscorea opposita Thunb.*, *Poria cocos (Schw.) Wolf*, and *Alisma orientale* (Sam.) *Juzep*.), and sourced exclusively from Good Agricultural Practice (GAP)-certified cultivation bases. Our previous study identified over 280 characteristic metabolites of the compound botanical drug Liuwei Dihuang Pills via LC-MS/MS detection, and further screened 30 candidate components with potential anti-AD pharmacological effects, namely, catalpol, loganin, morroniside, paeonol, paeoniflorin, pachymic acid, alisol A, alisol B, diosgenin, geniposide, rehmannioside C/D, and so on (Song et al., 2022a). (Supplementary Table S1).

The key reagents used in this study were primary antibodies, including the following: Amyloid-beta (Aβ, batch number: ab201060, Abcam), Peroxisome Proliferator-Activated Receptor Gamma Coactivator 1-Alpha (PGC-1α, batch number: PA5-3,802, Thermo Fisher Scientific), Mitofusin 2 (MFN2, batch number: 12186-1-AP, Proteintech), Fission 1 (FIS1, batch number: 10956-1-AP, Proteintech), Microtubule-Associated Protein 2 (MAP2, batch number: 8707T, Cell Signaling Technology (CST)), Postsynaptic Density Protein 95 (PSD-95, batch number: ab18258, Abcam), Synapsin (SYN, batch number: ab32127, Abcam), Insulin Receptor (InsR, batch number: gtx101136, GeneTex), Phosphorylated Insulin Receptor (p-InsR, batch number: 2386S, CST), Insulin Receptor Substrate 1 (IRS-1, batch number: 2382S, CST), Phosphorylated Protein Kinase B (p-AKT, batch number: ab38449, Abcam), Protein Kinase B (Akt, batch number: 108Y-1, Bioworld), Glycogen Synthase Kinase-3 Beta (GSK-3β, batch number: 27C10, CST), Phosphorylated Glycogen Synthase Kinase-3 Beta (p-GSK-3β, batch number: D3A4, CST), Glyceraldehyde-3-Phosphate Dehydrogenase (GAPDH, batch number: F057302, Sangon Biotech), and β-actin (batch number: AB0035, Biosharp). All above items are antibody reagents rather than small-molecule chemical compounds; complete product certificates and reagent information have been archived in our laboratory, and full reagent documents are available for provision if required.

### 2.2 Animals and drug treatment

Five-month-old male *APP* (K670N/M671L, Swedish)*/PSEN1ΔE9* (*APP/PS1*) transgenic mice (body weight: 20–30 g) and age- and sex-matched wild-type (WT) C57BL/6J littermates (negative controls) were obtained from the Model Animal Research Center of Nanjing University (Nanjing, Jiangsu Province, China). The facility holds an experimental animal use license (SCXK (Jiangsu) 2021–0,006). Mice were housed in a specific pathogen-free (SPF) facility under standardized environmental conditions: temperature maintained at 22 °C ± 2 °C, relative humidity at 60% ± 5%, and a 12 h light/12 h dark cycle (lights on at 08:00, lights off at 20:00). All animals had *ad libitum* access to autoclaved standard chow and ultrapure water filtered through a 0.22-μm membrane system.

Prior to intervention, mice underwent a 7-day acclimatization period to minimize environmental stress and associated physiological confounders. The experimental protocol was reviewed and approved by the Institutional Animal Care and Use Committee (IACUC) of Henan University of Chinese Medicine (Approval No.: DWLLGZR202202147), and all procedures strictly adhered to the National Institutes of Health (NIH) Guide for the Care and Use of Laboratory Animals (8th edition, 2011) and the ARRIVE 2.0 guidelines.

*APP/PS1* and age/sex-matched WT C57BL/6J mice were randomized into four groups (n = 10/group; sample size determined from preliminary tests and published pharmacological data for statistical power (Song et al., 2022a; Jia et al., 2022; Song et al., 2022b; Ding et al., 2024)): (1) control group (WT mice, saline gavage); (2) model group (*APP/PS1* mice, saline); (3) LWDHP group (*APP/PS1* mice, 0.675 g/kg/day (Jia et al., 2022; Song et al., 2022b; Ding et al., 2024), orally); (4) memantine group (positive group, *APP/PS1* mice, 3 mg/kg/day, orally). The dose of 0.675 g/kg/d was determined as the dose group via human-mouse body surface area conversion, equivalent to 3.75 g/kg/day crude drug. All interventions were delivered once daily by oral gavage for eight consecutive weeks. Vehicle-treated groups received equivalent volumes of 0.9% saline on the same schedule to ensure procedural consistency and minimize handling-related confounders.

### 2.3 Behavior experiments

Behavioral assessments were conducted during the final 5 days of the 8-week intervention period. In the Morris water maze (MWM) test, mice were trained over five consecutive days with four trials per day. For each trial, mice were gently placed into the circular pool (diameter: 120 cm; wall height: 50 cm) facing the inner wall at one of four pseudorandomized starting positions (1, 2, 3, 4), and the latency to locate and mount the submerged platform was recorded. A maximum trial duration of 60 s was imposed; mice failing to find the platform within this interval were guided to it and allowed to remain there for 15 s. On day 6 (probe trial), the platform was removed, and each mouse was released from the same starting position used on day 1 (3 quadrant). The time spent in the target quadrant (i.e., the quadrant formerly containing the platform) and the number of platform crossings (defined as complete passes over the former platform location with the mouse’s center point crossing a 10-cm-diameter virtual zone) were recorded over a 60-s testing period.

### 2.4 Transmission electron microscopy

Freshly dissected brain tissues were fixed by immersion in 2.5% glutaraldehyde prepared in 0.1 M sodium cacodylate buffer (pH 7.4) for 2 h at 4 °C, followed by post-fixation in 1% osmium tetroxide in the same buffer for 60 min at 4 °C. Tissues were then dehydrated through a graded ethanol series (30%, 50%, 70%, 90%, and 100% × 2), transitioned to propylene oxide, and embedded in Epon 812 resin. Ultrathin sections (70–90 nm) were cut using an ultramicrotome, mounted on copper grids, and stained with 2% uranyl acetate (aqueous, 15 min) followed by Reynolds’ lead citrate (5 min). Specimens were examined using a JEOL JEM-1400 transmission electron microscope operated at 100 kV.

### 2.5 Immunofluorescence assay

Paraffin sections prepared in Section 2.4 were dewaxed with xylene, rehydrated through a graded ethanol series, subjected to antigen retrieval (if required), blocked with 5% skim milk in PBS for 1 h at room temperature, and incubated overnight at 4 °C with primary antibody against phosphorylated tau (pSer404; 1:500 dilution). After washing, sections were incubated for 1 h at room temperature with Alexa Fluor 594-conjugated goat anti-mouse IgG (H + L) secondary antibody (1:1,000 dilution). Nuclei were counterstained with DAPI (1 μg/mL, 5 min). Immunofluorescence images were acquired using a fluorescence microscope equipped with appropriate filter sets.

### 2.6 Western-blot assay

Hippocampal and cortical tissues from each experimental group were homogenized on ice in RIPA lysis buffer (containing 1× protease and phosphatase inhibitor cocktails) using a pre-chilled mechanical tissue grinder. Homogenates were incubated on ice for 30 min with periodic vortexing, followed by centrifugation at 12,000 × g for 15 min at 4 °C. Supernatants containing total soluble protein were collected, and protein concentrations were quantified using the BCA assay according to the manufacturer’s instructions. Equal amounts of protein (30 μg per lane) were resolved by SDS-PAGE and transferred onto PVDF membranes. Membranes were blocked with 5% (w/v) non-fat dry milk in TBST for 1 h at room temperature, then incubated overnight at 4 °C with primary antibodies against pSer404-tau (1:1000), insulin receptor (INSR; 1:1000), IRS-1 (1:1000), pSer473-AKT (1:1000), total AKT (1:1000), pSer9-GSK-3β (1:1000), and total GSK-3β (1:1000). After three 10-min washes with TBST, membranes were incubated for 1 h at room temperature with HRP-conjugated secondary antibodies (goat anti-rabbit or goat anti-mouse IgG, 1:8000). Following final TBST washes, immunoreactive bands were visualized using enhanced chemiluminescence (ECL) substrate and captured with a digital imaging system. β-Actin (1:5000) served as the loading control, and band intensities were quantified using ImageJ software. All data were normalized to β-actin and expressed relative to the control group.

### 2.7 Statistical analysis

All experimental data were collected and organized using Microsoft Excel (Version 2021) and statistically analyzed via GraphPad Prism 8.0 software (GraphPad Software, Inc., United States), with results expressed as mean ± standard deviation (mean ± SD). Differences among multiple groups were assessed by one-way analysis of variance (one-way ANOVA) followed by Tukey’s post-hoc test for pairwise comparisons, and a two-tailed *P* < 0.05 was considered statistically significant. Sample size calculations were performed beforehand to ensure sufficient statistical power, and all experiments were conducted in triplicate with independent biological replicates (n ≥ 3 per group for animal experiments). This design minimizes random variation and guarantees the reliability and reproducibility of the results.

## 3 Results

### 3.1 Liuwei Dihuang pills mitigates the cognitive deficits of *APP/PS1* mice

**FIGURE 1:**
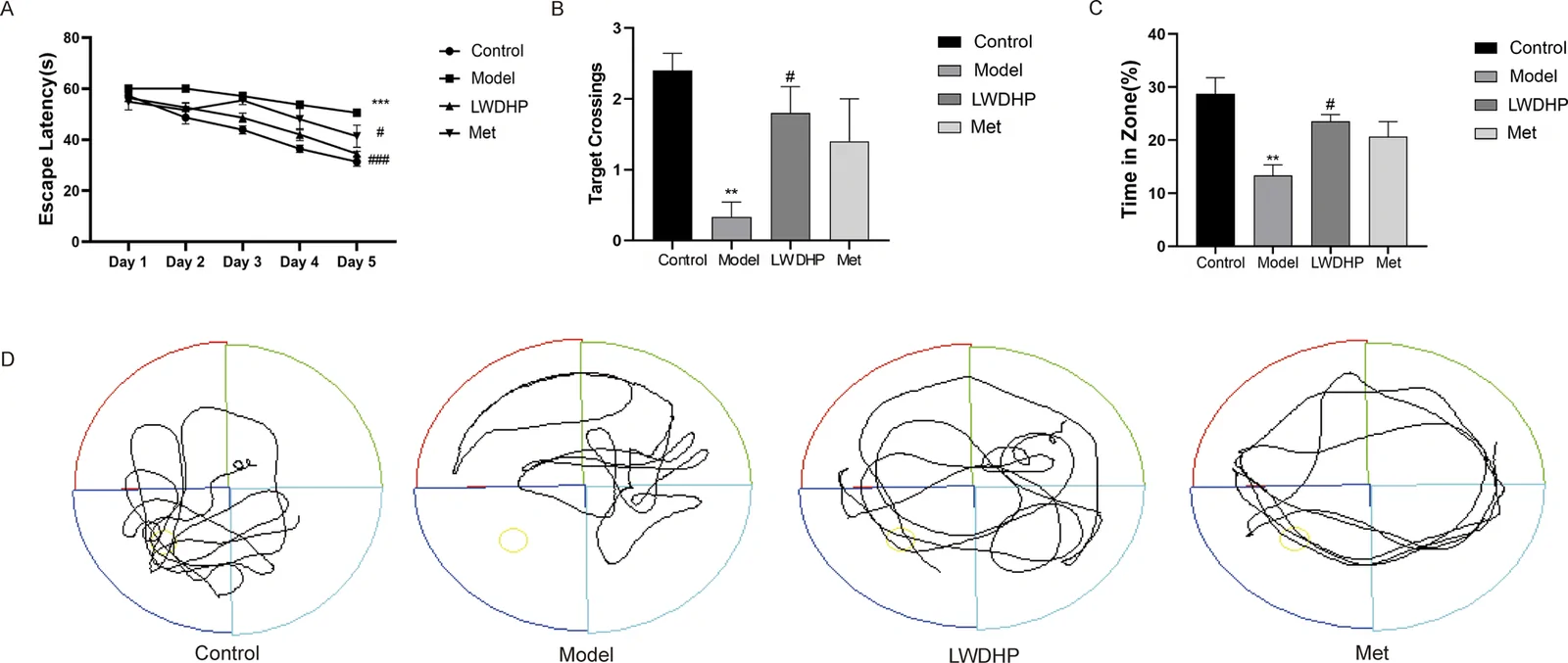
The effect of LWDHP on learning and memory of *APP/PS1* mice. **(A)** The escape latency to the platform during the training trails in a MWM. **(B)** Time spent in target quadrant in the probe test. **(C)** Target (platform) entries in the probe test. **(D)** Representative track images of mice in the probe test. Met served as a positive control. Data are means ± SEM (n = 10). Compared to control group **P ≤ 0.01, Compared to model group ^#^ *P* ≤ 0.05.

To investigate whether LWDHP ameliorates cognitive deficits in *APP/PS1* transgenic mice, spatial learning and memory were assessed using the morris water maze (MWM). During the hidden-platform acquisition phase (days 1–5), both escape latency and total swimming distance exhibited progressive reduction across training days in all groups, indicating intact procedural learning capacity. However, the model group showed significantly prolonged escape latency and increased total swimming distance compared with the control group on days 4 and 5 (*P* < 0.05), confirming robust spatial learning impairment. Importantly, no abnormalities in swimming posture, thigmotaxis, or locomotor activity were observed across any group, as verified by representative swim path trajectories and velocity analysis-thereby excluding motor confounds as contributors to the observed cognitive deficits. In the 60-s probe trial (day 6), the model group spent significantly less time in the target quadrant and exhibited fewer platform crossings than the controls (*P* < 0.05), indicative of impaired spatial reference memory. Treatment with LWDHP (0.675 g/kg/day) or memantine hydrochloride (3 mg/kg/day) significantly improved both acquisition performance (reduced latency and distance) and probe trial outcomes (increased target quadrant time and platform crossings) relative to the vehicle-treated the model group (*P* < 0.05). Notably, no statistically significant differences were detected between the LWDHP and memantine groups across all behavioral endpoints *(P* > 0.05), suggesting comparable efficacy (Figures 1A–D). These findings collectively demonstrate that daily oral administration of LWDHP at 0.675 g/kg effectively rescues spatial learning and memory deficits in *APP/PS1* transgenic mice.

### 3.2 Liuwei dihuang pills affect Aβ deposition and neurofibrillary in the brain of *APP/PS1* mice

**FIGURE 2:**
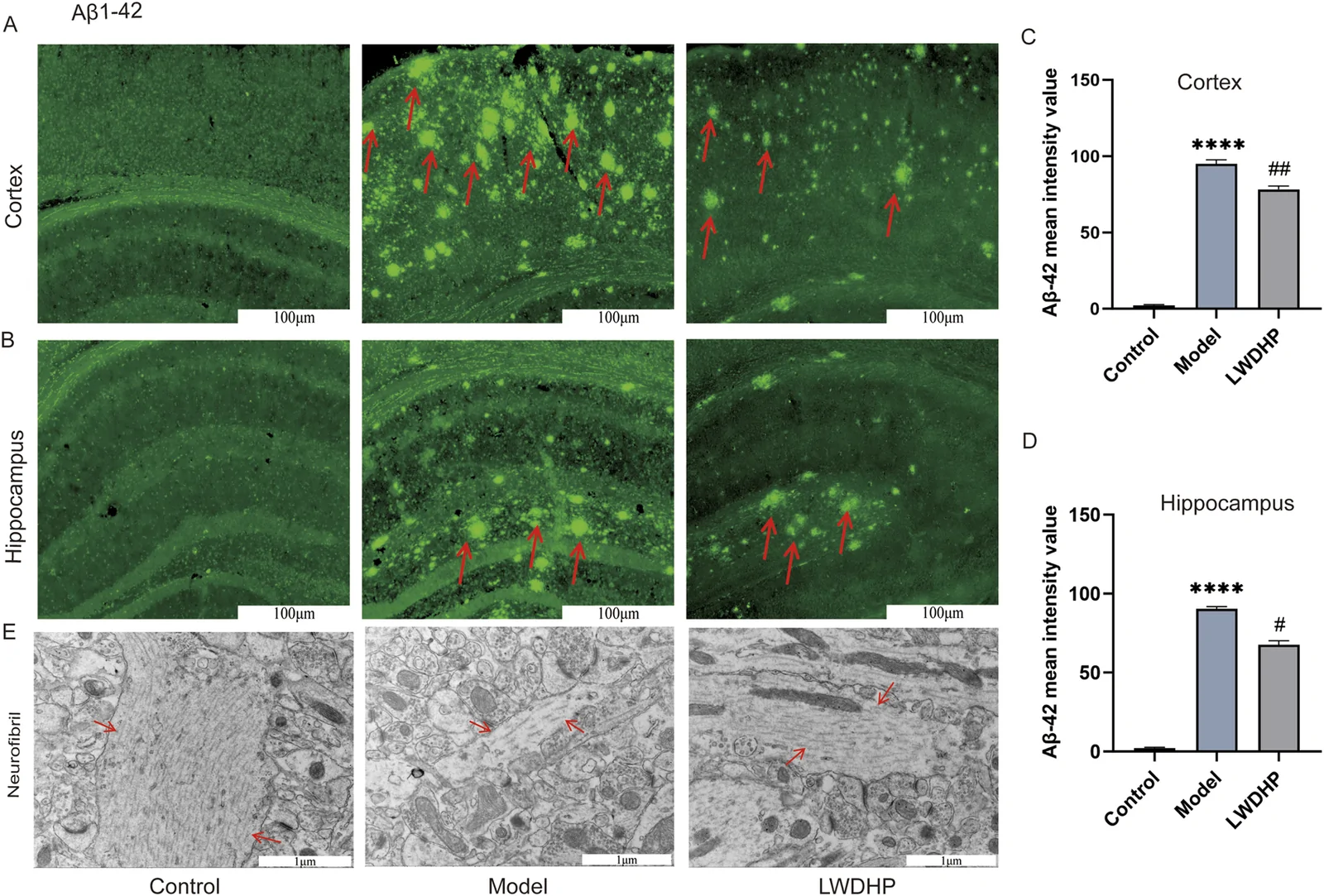
**(A)** The expression of Aβ plaques in the cortex of each group. **(B)** The expression of Aβ plaques in the hippocampus of each group. **(C)** Quantitative analysis of Aβ plaques in cortex. **(D)** Quantitative analysis of Aβ plaques in hippocampus. **(E)** The expression of neurofibrillary in hippocampus in each group. The red arrow indicates the Aβ plaques (Figure 2A,B) or the neuronal fibrils (Figure 2E). Data are means ± SEM (n = 3). Compared to control group ***P ≤ 0.001; Compared to model group ^#^ *P* ≤ 0.05, ^##^ *P* ≤ 0.01.

To further evaluate the effects of LWDHP on AD-related pathological hallmarks, we assessed Aβ_1-42_ plaque deposition via immunofluorescence staining and examined neuronal ultrastructural changes using transmission electron microscopy (TEM). As shown Figures 2A–D, Aβ_1-42_ immunoreactivity was sparse and faint in the control group. In contrast, the model group displayed widespread, intensely immunoreactive Aβ_1-42_ plaques in the cortex and the hippocampus, indicative of robust amyloid pathology. After LWDHP treatment, the number and intensity of Aβ_1-42_ plaques were markedly reduced compared to the model group, demonstrating a significant inhibitory effect on Aβ deposition. TEM analysis of hippocampal neuronal ultrastructure revealed marked pathological changes in the model group (Figure 2E). The neurofibrillary of the control group showed well-organized, densely and parallelly arranged neurofibrils. Compare to control group, prominent disruptions of neurofibrillary architecture of the model group, including disorganized microtubule arrays, fragmented neurofibrils, reduced density, and widened inter-fibrillar spaces. Notably, LWDHP treatment markedly attenuated these ultrastructural abnormalities: neurofibrils in the LWDHP group exhibited improved structural organization and increased packing density relative to the model group. These results indicate that LWDHP effectively attenuates Aβ_1-42_ plaque formation and preserves nneurofibrillary architecture in *APP/PS1* mice, suggesting a protective effect against AD-related neuropathology.

### 3.3 Liuwei Dihuang pills restored neuronal mitochondrial homeostasis in *APP/PS1* mice

**FIGURE 3:**
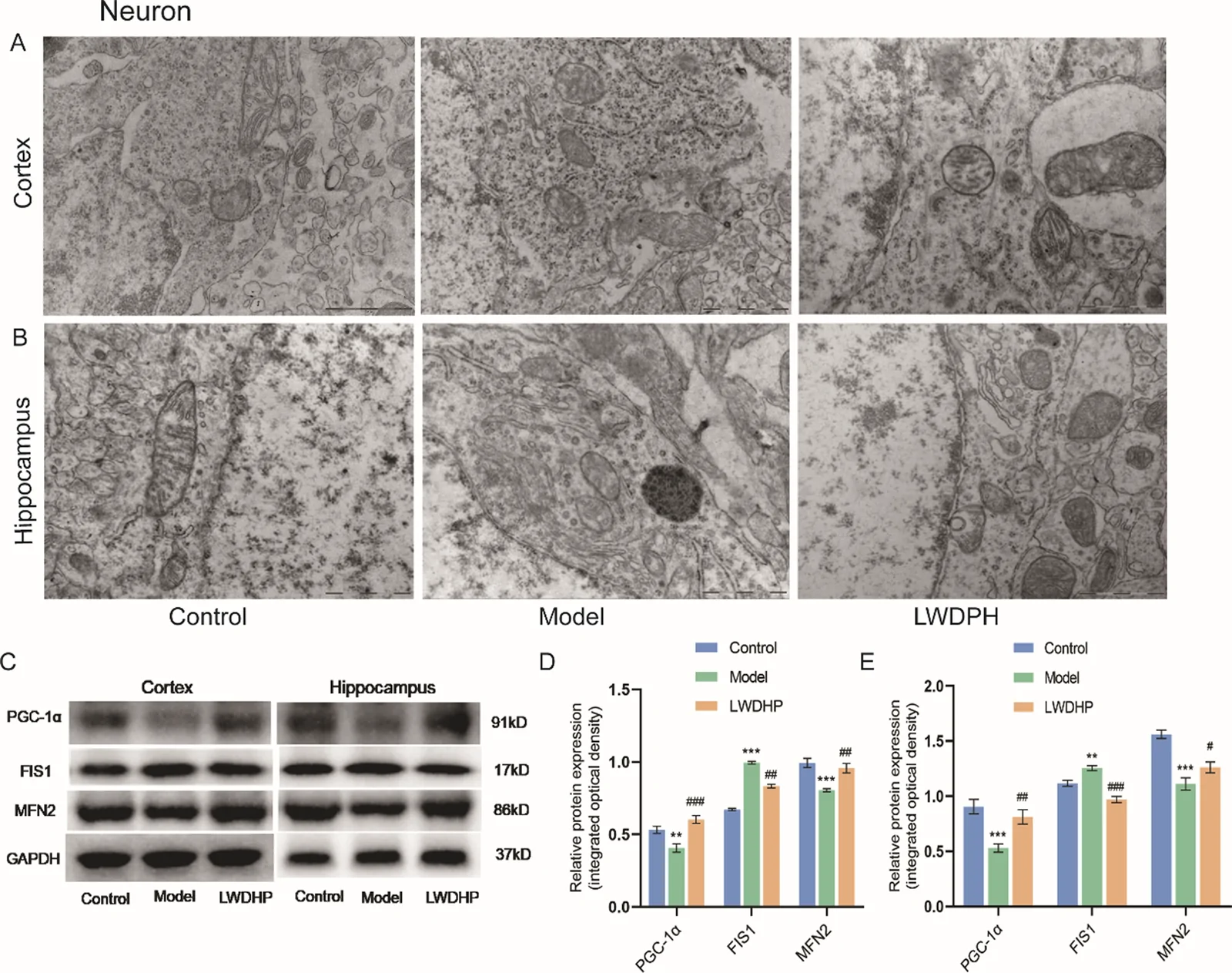
Liuwei Dihuang Pills restored neuronal mitochondrial homeostasis in *APP/PS1* mice. **(A)** Neuron ultrastructure in the cortex of each group. **(B)** Neuron ultrastructure in the hippocampus of each group. **(C)** The protein exprssion of PCG-1α, FIS1 and MFN2 in each group. **(D)** Quantitative histogram of PCG-1α, FIS1 and MFN2 in the cortex. **(E)** Quantitative histogram of PCG-1α, FIS1 and MFN2 in the hippocampus. The red arrow indicates mitochondrion. Data are means ± SEM (n = 3). Compared to control group **P ≤ 0.01, ***P ≤ 0.001; Compared to model group ^#^ *P* ≤ 0.05, ^##^ *P* ≤ 0.01, ^###^ *P* ≤ 0.001.

Using transmission electron microscopy, we examined the neuronal ultrastructure in the cerebral cortex and hippocampus (Figures 3A,B). Neurons from the control group showed normal nuclear architecture, along with mitochondria that were closely arranged and largely oval or elongated in shape, displaying tightly aligned and uninterrupted cristae. The rough endoplasmic reticulum remained morphologically intact across both cortical and hippocampal regions, with ribosomes evenly anchored to its membranes. By comparison, neurons in the model group displayed severe subcellular abnormalities in these same brain areas: condensed and shrunken nuclei, swollen mitochondria with disintegrated, fragmented, or absent cristae, intramitochondrial vacuoles, and markedly distended RER profiles lacking surface-bound ribosomes. Treatment with LWDHP markedly ameliorated the observed ultrastructural abnormalities: mitochondrial swelling and cristae fragmentation were significantly attenuated; the majority of mitochondria exhibited well-preserved cristae architecture, and intracellular organelles remained abundant and spatially organized, with only minor residual mitochondrial alterations evident (Figures 3A,B). Concurrently, we evaluated the effects of LWDHP on key regulators of mitochondrial dynamics. Specifically, the fusion-promoting proteins PGC-1α and MFN2, and the fission-associated protein FIS1. Quantitative Western blot analysis revealed that, relative to control group, *APP/PS1* mice exhibited significantly reduced cortical and hippocampal expression levels of both PGC-1α and MFN2, whereas FIS1 protein abundance was markedly elevated. Notably, LWDHP treatment restored the imbalance: it upregulated PGC-1α and MFN2 expression while concurrently suppressing FIS1 levels, thereby promoting a shift toward enhanced mitochondrial fusion and improved fusion-fission equilibrium (*P* < 0.05 or *P* < 0.0001, Figures 3C–E). These results indicate that LWDHP can effectively restore the disrupted mitochondrial dynamic balance in the *APP/PS1* mouse model and reconstruct the damaged mitochondrial biogenesis process to protect neurons.

### 3.4 Liuwei Dihuang pills ameliorates synaptic plasticity impairment in the brain of *APP/PS1* mice

**FIGURE 4:**
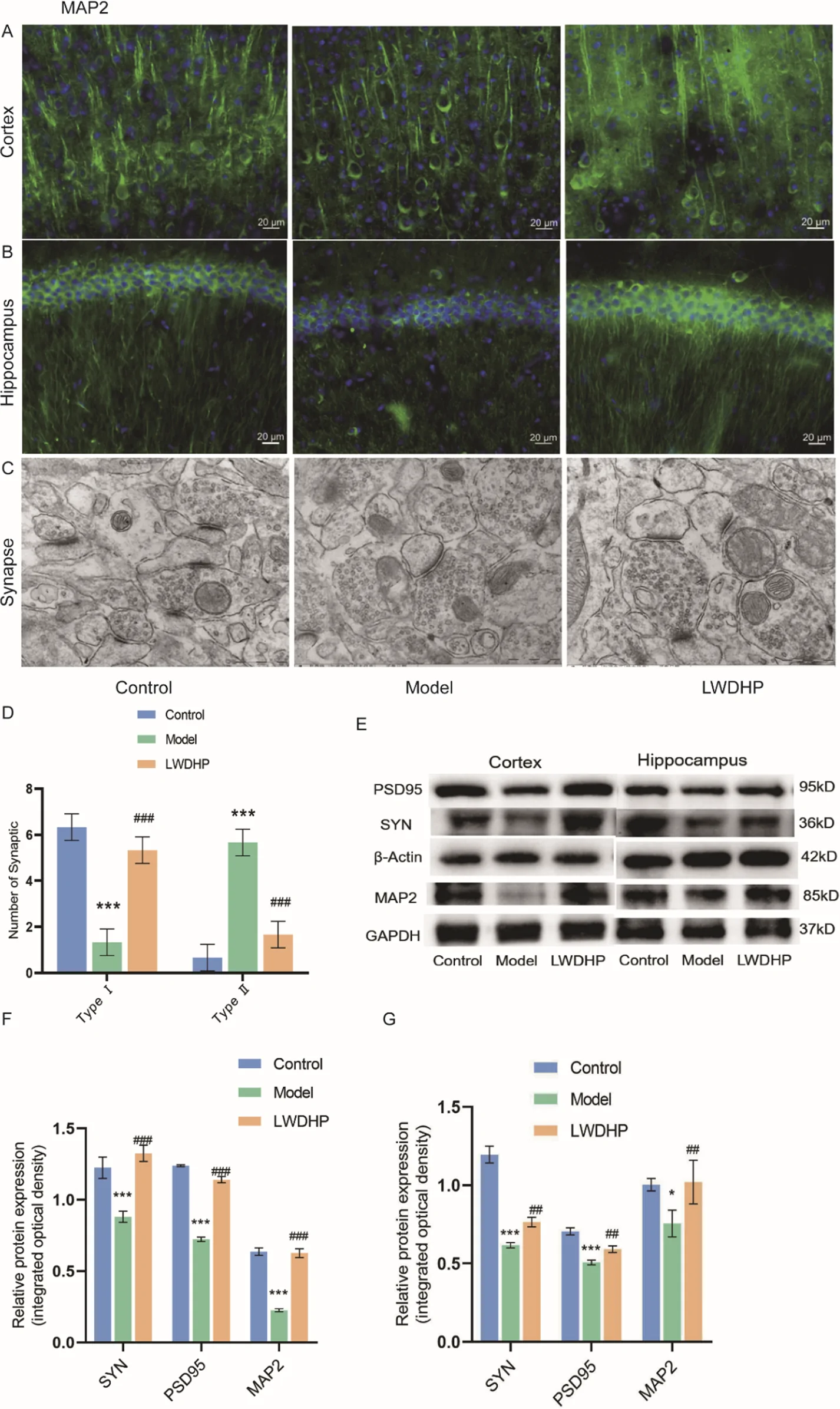
LWDHP restores structural synaptic integrity in neurons of the brains in *APP/PS1* mice. **(A)** The expression of MAP2 in the cortex of each group. **(B)** The expression of MAP2 in the hippocampus of each group. **(C)** The synapses of hippocampal neuron (Scale bar = 500 nm, The red arrow indicates the type I synapses, and the green arrow indicates the type II synapses). **(D)** The relative value of type I synapses and the relative value of type II synapses. **(E)** The protein exprssion of PSD95, SYN and MAP2 in the cortex and hippocampus of each group. **(F)** Quantitative histogram of PSD95, SYN and MAP2 in the cortex. **(G)** Quantitative histogram of PSD95, SYN and MAP2 in the hippocampus. Data are means ± SEM (n = 3). Compared to control group *P ≤ 0.05, ***P ≤ 0.001; Compared to model group ^##^ *P* ≤ 0.01, ^###^ *P* ≤ 0.001.

Synaptic plasticity impairment represents a core pathological mechanism underlying the progressive decline in learning and memory functions observed in AD (Lai et al., 2025). In this study, we systematically evaluated dendritic integrity and synaptic ultrastructure in the cortex and hippocampus of *APP/PS1* model mice and the control. MAP2 immunofluorescence analysis revealed significant reductions in dendritic density in both cortical and hippocampal regions of AD model mice relative to control mice (Figures 4A,B). Consistent with these morphological alterations, transmission electron microscopic observation demonstrated a prominent reduction in mature type I synapses accompanied by a robust elevation of immature type II synapses in the AD model group. Notably, LWDHP administration effectively reversed these abnormal structural changes, restoring dendritic complexity and the proportion of mature synapses (Figures 4C,D). Notably, LWDHP effectively ameliorated these structural abnormalities, restoring dendritic complexity and synaptic maturation profiles toward WT levels. Consistent with these morphological improvements, LWDHP treatment significantly reversed the downregulation of key synaptic proteins—including microtubule-associated protein 2 (MAP2), postsynaptic density protein 95 (PSD-95), and synaptophysin (SYN) in the cortex and hippocampus of AD mice. Collectively, these findings indicate that LWDHP exerts neuroprotective effects in AD by preserving dendritic architecture and supporting synaptic plasticity.

### 3.5 Liuwei dihuang pills modulate IRS-1/AKT/GSK-3β signaling in the brain of *APP/PS1* mice

**FIGURE 5:**
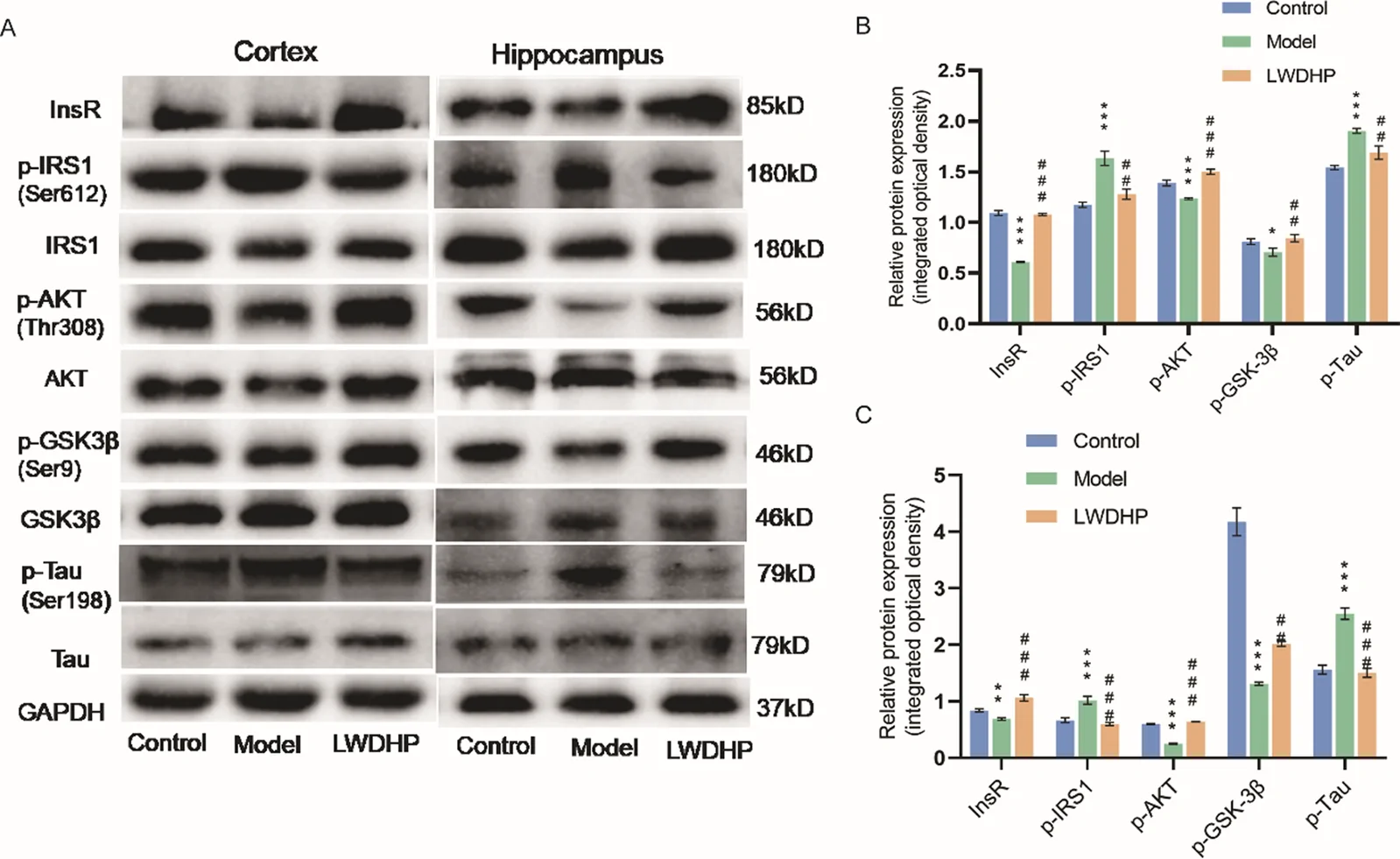
LWDHP modulate IRS1/AKT/GSK-3β signaling pathway related proteins in the brain of *APP/PS1* mice. **(A)** The protein expression of IRS1/AKT/GSK-3β signaling pathway related proteins in the cortex and hippocampus of each group. **(B)** Quantitative histogram of IRS1/AKT/GSK-3β signaling pathway related proteins in the cortex. **(C)** Quantitative histogram of IRS1/AKT/GSK-3β signaling pathway related proteins in the hippocampus. Data are means ± SEM (n = 3). Compared to control group *P ≤ 0.05, **P ≤ 0.01, ***P ≤ 0.001; Compared to model group ^##^ *P* ≤ 0.01, ^###^ *P* ≤ 0.001.

To investigate the therapeutic potential of LWDHP in ameliorating cerebral insulin signaling in AD, we quantified the expression and site-specific phosphorylation of core components within the IRS-1/AKT/GSK-3β signaling in the cerebral cortex and hippocampus of *APP/PS1* transgenic mice using quantitative immunoblotting. The protein expression of insulin receptor (InsR), phospho-IRS-1 (Ser612), total IRS-1, phospho-AKT (Thr308), total AKT, phospho-GSK-3β (Ser9), total GSK-3β, phospho-Tau (Ser198), and total Tau are presented in Figure 5. Compared with Control group, InsR protein expression was significantly reduced in the cerebral cortex and hippocampus of Model group (*P* < 0.01, *P* < 0.05). Concomitantly, AKT at Thr308 (p-AKT/AKT ratio, *P* < 0.01), and GSK-3β at Ser9 (p-GSK-3β/GSK-3β ratio, *P* < 0.05, *P* < 0.001) were all significantly attenuated,but phosphorylation of IRS-1 at Ser612 (*P*-IRS-1/IRS-1 ratio, *P* < 0.001) was significantly increased. These rsults suggest impaired upstream insulin signaling and excessive activation of GSK-3β. In line with this observation, Tau phosphorylation at Ser198 (p-Tau/Tau ratio) was significantly elevated (*P* < 0.001, *P* < 0.01). LWDHP treatment effectively counteracted these deficits: InsR expression was restored (*P* < 0.01); p-IRS-1/IRS-1 ratios was reduced, p-AKT/AKT ratios was increased significantly (*P* < 0.001 and *P* < 0.01, respectively); and inhibitory phosphorylation of GSK-3β at Ser9 was normalized (*P* < 0.01), leading to a significant reduction in p-Tau/Tau levels relative to the model group (*P* < 0.01). Collectively, these findings demonstrate that LWDHP restore cerebral insulin sensitivity and attenuate Tau hyperphosphorylation in both the cerebral cortex and hippocampus of *APP/PS1* transgenic mice, which effects that are mechanistically linked, at least in part, to the functional modulation of the IRS-1/AKT/GSK-3β signaling cascade.

## 4 Discussion

A growing body of clinical and experimental evidence underscores a robust pathophysiological link between Alzheimer’s disease (AD) and type 2 diabetes mellitus (T2DM^)^ (Lv et al., 2025; Kciuk et al., 2024; Donat Ergin et al., 2024). The shared pathogenic mechanisms of these two diseases involve abnormal amyloid protein aggregation, insulin deficiency and resistance, inflammatory responses, oxidative stress, and impaired autophagy (Lv et al., 2025; Kciuk et al., 2024; Donat Ergin et al., 2024; Okabayashi et al., 2015). From the perspective of Traditional Chinese Medicine (TCM), “kidney yin deficiency” is regarded as the core common pathogenesis underlying both disorders. The therapeutic strategy of tonifying the kidney and replenishing essence not only exerts neuroprotective effects in AD treatment but also effectively alleviates symptoms in patients with type 2 diabetes by nourishing yin and tonifying the kidney (Lv et al., 2025). Liuwei Dihuang Pills (LWDHP), a classic TCM formula for nourishing kidney yin, was initially proposed by the renowned physician Qian Yi of the Song Dynasty in his work “Direct Decisions on Pediatric Medication”. LWDHP exerts characteristic pharmacological effects of nourishing yin and tonifying the kidney, and is a commercial compound botanical drug widely applied in TCM clinical practice for the treatment of diseases related to kidney yin deficiency (Song et al., 2022a). Our previous studies have confirmed that LWDHP can protect neurons in AD by inhibiting inflammatory responses (Song et al., 2022a), however, its regulatory effects on the insulin signaling pathway and the underlying molecular mechanisms remain incompletely elucidated.

In the present study, the Morris Water Maze (MWM) test demonstrated that LWDHP significantly ameliorated spatial learning and memory deficits in *APP/PS1* mice. This improvement in cognitive function was accompanied by reduced amyloid-β (Aβ) deposition and alleviated neurofibrillary pathology, which is consistent with the findings of our previous research (Song et al., 2022a; Yuan et al., 2023). Aβ aggregation and abnormal tau hyperphosphorylation are well-recognized core pathological features of AD, and these processes are closely associated with neuronal loss, synaptic dysfunction, and progressive cognitive decline (Nasreddine et al., 2023; Basaly et al., 2021). The ability of LWDHP to reduce Aβ accumulation may be closely related to its regulatory effects on mitochondrial function and insulin signaling, as accumulating evidence has shown that mitochondrial impairment and insulin resistance can promote Aβ production and aggregation (Chornenkyy et al., 2019). Furthermore, our study observed that LWDHP enhanced the density and structural integrity of nerve fibers, which may further facilitate the recovery of cognitive function by improving interneuronal communication and signal transmission.

Mitochondrial dysfunction is widely acknowledged as an early and pivotal pathological event in AD, occurring prior to the formation of Aβ plaques and neurofibrillary tangles (NFTs) (Lai et al., 2025; Hussain et al., 2025). Mitochondrial dysfunction is associated with peroxisome proliferator-activated receptor γ coactivator 1α (PGC-1α) and mitochondrial dynamics regulators such as Mitofusin 2 (MFN2) and Fission 1 (FIS1). PGC1α serves as a master regulator of mitochondrial biogenesis, oxidative phosphorylation (OXPHOS), and antioxidant defense systems (Katsouri et al., 2016; Hou et al., 2022). In the brains of AD patients, the transcriptional activity and expression level of PGC1α are significantly downregulated, which directly impairs mitochondrial DNA replication, reduces OXPHOS efficiency, and promotes the overproduction of reactive oxygen species (ROS) (Katsouri et al., 2016; Hou et al., 2022). MFN2 acts as a key mediator of mitochondrial fusion, which is essential for maintaining the integrity of the mitochondrial network and facilitating functional complementation between individual mitochondria (R et al., 2024). FIS1 is a critical driver of mitochondrial fission, and its overactivation leads to mitochondrial fragmentation, a typical hallmark of mitochondrial dysfunction in AD (R et al., 2024; Lei et al., 2023). This cascade of events not only disrupts mitochondrial structural integrity but also creates a pro-oxidative microenvironment, thereby exacerbating neuronal damage and accelerating AD progression (Lai et al., 2025; Hussain et al., 2025). Consistent with these findings, our study demonstrated that LWDHP reversed mitochondrial ultrastructural damage in APP/PS1 mice, including reducing mitochondrial swelling and fragmentation. Moreover, LWDHP upregulated the expression of mitochondrial function-related markers such as PGC1α and Mfn2, while downregulating FIS1 expression. In AD models, the downregulation of PGC1α disrupts this balance, resulting in increased FIS1-mediated fission and decreased MFN2-mediated fusion; this ultimately leads to fragmented mitochondria with impaired energy production and enhanced ROS release (Zhu et al., 2025; Katsouri et al., 2016). Emerging evidence indicates that PGC1α can directly upregulate MFN2 expression and downregulate FIS1 expression through transcriptional regulation, thereby maintaining the dynamic balance of mitochondria (Zhu et al., 2025). However, we did not investigate the regulatory relationship between PGC-1, MFN2, and FIS1 in depth in the paper, which requires further validation. Notably, overexpression of MFN2 has been shown to rescue mitochondrial fragmentation and mitigate tau-induced neurotoxicity, while inhibition of FIS1 can alleviate Aβ-mediated neuropathological changes, highlighting the therapeutic potential of targeting these regulators of mitochondrial dynamics in AD (Zhu et al., 2025; Katsouri et al., 2016). We confirmed that LWDHP restores the balance of mitochondrial fusion/fission and promotes mitochondrial biogenesis, thereby improving mitochondrial function and reducing neuronal damage in AD. Interestingly, mitochondria are the main sites of cellular energy production, and impaired mitochondrial function leads to insufficient adenosine triphosphate (ATP) supply-an essential prerequisite for the normal phosphorylation and activation of key molecules in the insulin signaling pathway. This indicates that the insulin signaling pathway is closely implicated in the pathogenesis of AD.

AD has been increasingly recognized as “type 3 diabetes” due to the presence of brain insulin resistance and impaired insulin signaling (Lanzillotta et al., 2024; Kciuk et al., 2024). Insulin plays a vital role in regulating neuronal survival, synaptic plasticity, and cognitive function, and its downstream signaling pathway (IRS1/AKT/GSK3β) is frequently disrupted in AD (Bagheri-Mohammadi et al., 2022; Tyagi and Pugazhenthi, 2021). Our previous studies have validated that Radix Rehmanniae Praeparata (Shu Dihuang), a classic yin-nourishing herbal medicine with proven hypoglycemic and memory-improving properties, exerts prominent neuroprotective effects in ICV-STZ-induced AD mice by regulating the INSR/IRS-1/AKT/GSK-3β signaling pathway and remodeling intestinal microbiota homeostasis (Su et al., 2023). These findings are highly consistent with current research consensus that numerous food-medicine homologous Chinese medicines and their derived bioactive components possess potent neuroprotective activities, further supporting the therapeutic potential of TCM homologous ingredients for AD intervention (Ye et al., 2025; Law and Au, 2026). Experimental studies have demonstrated reduced levels of insulin, insulin receptor (IR) mRNA, and insulin receptor substrate (IRS)-associated p-PI3K/AKT in the brains of AD patients (Tyagi and Pugazhenthi, 2021; Lauretti et al., 2020). Consistent with these reports, our study observed decreased InsR levels, AKT (Thr308), and GSK3β (Ser9), but phosphorylation of IRS-1 (Ser612) and Tau was increased in the brain of in *APP/PS1* mice. This demonstrates notable dysfunction of central insulin signaling in Alzheimer’s disease models. Peripheral insulin resistance is closely pathologically associated with central insulin signaling disorder. In the present study, we did not measure insulin levels in blood or cerebrospinal fluid (CSF). Further investigations into the regulatory effects of LWDHP on the peripheral-central insulin axis in AD will be of great significance in future work.

GSK3β is a key kinase that promotes tau hyperphosphorylation, In addition to regulating tau phosphorylation, overactivated GSK3β can directly modulate the expression and function of synaptic proteins, thereby impairing synaptic transmission and plasticity (Lauretti et al., 2020). Our results support exactly this conclusion. Importantly, LWDHP treatment effectively restored the phosphorylation levels of these molecules, reinstating IRS1/Akt signaling and inhibiting GSK3β activation. Furthermore, LWDHP may further decrease NFTs formation. Central IRS1/AKT/GSK3β signaling pathway plays a critical role in maintaining neuronal survival, energy metabolism, and synaptic plasticity (Bagheri-Mohammadi et al., 2022; Tyagi and Pugazhenthi, 2021). GSK-3β not only phosphorylates MAP2 to destabilize dendritic microtubules and impair synaptic function by interfering with synaptic vesicle transport and dendritic structure maintenance, but also reduces PSD95 and SYN levels through transcriptional modulation or protein degradation, which further damages synaptic transmission and plasticity (Ansari et al., 2023)^.^ However, this conclusion still requires verification through further experimental studies.

Neuronal synaptic proteins MAP2, PSD95, and SYN are key markers of synaptic structure and function, and their expression levels are closely related to synaptic density and synaptic transmission efficiency. Our study confirmed that LWDHP treatment significantly increased the expression levels of MAP2, PSD95, and SYN in the hippocampus and cortex of AD model mice, which was closely related to the improvement of synaptic density and cognitive function. In AD, synaptic loss is an early and prominent pathological feature, accompanied by a significant decrease in the expression of MAP2, PSD95, and SYN (Ansari et al., 2023). Reduced expression of these synaptic proteins is tightly linked to PGC-1α dysfunction and insulin resistance (Katsouri et al., 2016; Bagheri-Mohammadi et al., 2022; Tyagi and Pugazhenthi, 2021). As noted above, impaired IRS-1/Akt/GSK-3β signaling aggravates the loss of synaptic proteins via modulating their phosphorylation and transcription (Bagheri-Mohammadi et al., 2022; Tyagi and Pugazhenthi, 2021; Su et al., 2023). In addition, PGC-1α deficiency triggers mitochondrial dysfunction and oxidative stress, which disrupt synaptic architecture and suppress the synthesis of synaptic proteins (Lai et al., 2025; Hussain et al., 2025). A variety of neuroprotective active ingredients from homologous Chinese medicines have been proven to improve synaptic structural integrity and synaptic transmission function by improving oxidative stress and metabolic disorders, which is consistent with the synaptic protective effect of LWDHP observed in this study (Ye et al., 2025). These regulatory effects of LWDHP on the PGC1α and IRS1/AKT/GSK3β pathways further contribute to the upregulation of synaptic proteins MAP2, PSD95, and SYN. These suggest that the restoration of insulin signaling by LWDHP can also enhance synaptic plasticity and neuronal survival, which further contributing to the improvement of cognitive function.

Our results indicate that LWDHP upregulate the expression of synaptic proteins PSD-95, Syn and MAP2, and improve synaptic plasticity. LWDHP may exert a synergistic regulatory effect on these pathways: upregulation of PGC1α by LWDHP improves mitochondrial function, which provides energy support for the activation of the IRS1/AKT/GSK3β pathway; in turn, activated AKT can further enhance PGC1α expression through a positive feedback mechanism, thereby forming a virtuous cycle that jointly protects neurons from damage (Hou et al., 2022; Lei et al., 2023). The cross-talk between PGC1α and the IRS1/AKT/GSK3β pathway could forms a complex regulatory network that modulates the progression of AD. Whether LWDHP modulates both pathways to exert synergistic effects against AD pathology remains unclear and requires further investigation. More sophisticated approaches, including single-cell sequencing and proteomics, should be applied to unravel the detailed mechanisms underlying LWDHP action. In addition, complementary *in vitro* cell assays and reverse validation are needed to strengthen the findings, which will further support its clinical applicationfor AD treatment.

In conclusion, our study demonstrates that LWDHP rescues cognitive deficits in *APP/PS1* mice by regulating multiple pathological pathways of AD, including reducing Aβ deposition and neurofibrillary pathology, protecting mitochondrial function, and restoring the IRS1/AKT/GSK3β insulin signaling pathway. These findings highlight the potential of LWDHP as a promising therapeutic agent for AD and provide preclinical pharmacological evidence supporting further translational research of this classic compound botanical drug.

## Data Availability

The original contributions presented in the study are included in the article/Supplementary Material further inquiries can be directed to the corresponding authors.
