# Supplementary material for: Liuwei Dihuang pills attenuates cognitive dysfunction in APP/PS1 mice through restoring mitochondrial homeostasis and activating the IRS/AKT/GSK3β insulin signaling pathway
Source: Front Pharmacol. 2026 Aug 20;17:1873606. doi: 10.3389/fphar.2026.1873606 (PMC13538836; doi:10.3389/fphar.2026.1873606)
Supplement: Supplementary file 1 [file Table1.pdf]

Supplementary Table 1. The Bioactive Components of LWDPH

	Mol ID	Molecule Name	MW	AlogP	OB (%)	Caco-2	BBB
Paeonia suffruticosa	MOL000103	PHB	138.13	1.17	30.15	0.39	0.21
	MOL000114	vanillic acid	168.16	1.15	35.47	0.43	0.09
	MOL001246	(1R)-(+)-Nopinone	138.23	1.52	57.86	1.23	1.6
	MOL001551	Trochol	442.8	6.31	15.48	0.84	0.35
	MOL001915	paeonoside	610.57	-2.22	3.47	-2.71	-3.73
	MOL009092	Pentagalloylglucose	940.72	3.69	3.01	-3.08	-4.17
	MOL001925	paeoniflorin_qt	318.35	0.46	68.18	-0.34	-0.73
	MOL002046	hexanoic acid	116.18	1.81	73.08	0.8	0.93
	MOL000211	Mairin	456.78	6.52	55.38	0.73	0.22
	MOL000219	BOX	121.12	0.76	31.55	0.54	0.84
	MOL000223	caffeic acid	180.17	1.37	25.76	0.21	-0.26
	MOL000263	oleanolic acid	456.78	6.42	29.02	0.59	0.07
	MOL002786	Apocynin	166.19	1.29	31.71	0.74	0.52
	MOL000359	sitosterol	414.79	8.08	36.91	1.32	0.87
	MOL003881	6-Hydroxycoumarin	162.15	1.63	15.8	0.73	0.47
	MOL000422	kaempferol	286.25	1.77	41.88	0.26	-0.55
	MOL000492	(+)-catechin	290.29	1.92	54.83	-0.03	-0.73
	MOL005090	oxypaeoniflorin_qt	334.35	0.2	19.4	-0.77	-1.28
	MOL000513	3,4,5-trihydroxybenzoic acid	170.13	0.63	31.69	-0.09	-0.54
	MOL000675	oleic acid	282.52	6.84	33.13	1.17	0.78
	MOL006869	WLN: QR CQ DV1	152.16	1.03	36.49	0.67	0.46
	MOL007002	paeonioflorin	480.51	-1.28	10.22	-1.69	-2.39
	MOL007003	benzoyl paeoniflorin	584.62	0.76	31.14	-1.35	-2.08
	MOL007006	oxypaeoniflorin	496.51	-1.55	12.98	-1.91	-2.7
	MOL003867	Paeonolide	460.48	-1.85	6.3	-1.42	-2.09
	MOL000719	methyl salicylate	152.16	1.42	42.55	1.05	1.1
	MOL000254	eugenol	164.22	2.55	56.24	1.35	1.32
	MOL007364	apiopaeonoside	460.48	-2.03	16.73	-1.79	-2.48
	MOL007367	paeonoside	328.35	-0.62	18.52	-0.86	-1.04
	MOL007368	4-O-methylpaeoniflorin	494.54	-0.87	25.71	-1.27	-1.7
	MOL007369	4-O-methylpaeoniflorin_qt	332.38	0.87	67.24	0.15	-0.15
	MOL007373	1-(2,3-dihydroxy-4-methoxyphenyl)ethanone	182.19	1.02	32.96	0.81	0.63
	MOL007374	5-[[5-(4-methoxyphenyl)-2-furyl]methylene]barbituric acid	312.3	2.05	43.44	0.09	-0.7
	MOL007377	mudanoside A	342.33	-1	13.39	-1.42	-1.67
	MOL007378	3-acetyl-4-hydroxybenzoic acid	180.17	0.91	37.58	-0.04	-0.17
	MOL007382	mudanpioside-h_qt 2	336.37	-0.03	42.36	-0.39	-0.96
	MOL007383	paeonidanin	492.57	-0.83	24.64	-1.28	-1.71

	MOL007384	paeonidanin_qt	330.41	0.92	65.31	-0.09	-0.59
	MOL000874	paeonol	166.19	1.29	28.79	0.93	0.84
	MOL000095	delta 7-stigmastenol	416.81	8.33	25.32	1.31	0.98
	MOL000098	quercetin	302.25	1.5	46.43	0.05	-0.77
Cornus officinalis	MOL000105	protocatechuic acid	154.13	0.9	25.37	0.1	-0.17
	MOL000118	(L)-alpha-Terpineol	154.28	2.42	48.8	1.39	1.72
	MOL000126	(-)-nopinene	136.26	2.93	44.84	1.8	2.12
	MOL001300	PEL	122.18	1.55	44.03	1.11	1.13
	MOL000131	EIC	280.5	6.39	41.9	1.16	0.9
	MOL001393	myristic acid	228.42	5.46	21.18	1.07	0.99
	MOL001398	Methyllinolenate	292.51	6.2	46.15	1.48	1.09
	MOL001494	Mandenol	308.56	6.99	42	1.46	1.14
	MOL001495	Ethyl linolenate	306.54	6.55	46.1	1.54	1.12
	MOL001599	α-cubebol	208.38	3.28	64.81	1.32	1.43
	MOL001600	copaene	204.39	4.17	29.47	1.81	2.04
	MOL001604	Linalool	170.28	1.43	49.37	0.86	0.74
	MOL001606	BB_NC-0668	204.39	4.75	35.57	1.83	2.02
	MOL001618	Pellitorin	223.4	3.96	23.81	1.36	1.1
	MOL001680	Loganin	390.43	-2.08	5.9	-1.48	-2.26
	MOL001683	morroniside_qt	244.27	-0.72	1.68	-0.6	-1.25
	MOL001696	Morusin	420.49	4.94	11.52	0.51	-0.22
	MOL001739	zoomaric acid	254.46	5.92	35.78	1.18	0.88
	MOL001746	ELD	281.54	6.23	31.2	1.26	1.07
	MOL001771	poriferast-5-en-3beta-ol	414.79	8.08	36.91	1.45	1.14
	MOL002343	tetrandrine	622.82	7.22	26.64	1.02	0.44
	MOL002373	gamma-tocopherol	416.76	9.93	15.62	1.58	1.12
	MOL000261	Myristicin	192.23	2.59	17.99	1.35	1.14
	MOL000263	oleanolic acid	456.78	6.42	29.02	0.59	0.07
	MOL001889	Methyl linolelaidate	294.53	6.64	41.93	1.46	1.11
	MOL001893	BU3	90.14	-0.14	34.87	0.19	-0.01
	MOL001906	Methylgallate	184.16	0.88	30.91	0.26	0.06
	MOL000207	Methyleugenol	178.25	2.81	73.36	1.47	1.41
	MOL000223	caffeic acid	180.17	1.37	25.76	0.21	-0.26
	MOL000027	alpha-Curcumene	202.37	5.34	4.68	1.93	1.99
	MOL002778	THZ	135.2	1.91	70.33	1.39	1.51
	MOL002850	butylated hydroxytoluene	220.39	4.85	40.02	1.75	1.8
	MOL002879	Diop	390.62	7.44	43.59	0.79	0.26
	MOL002883	Ethyl oleate (NF)	310.58	7.44	32.4	1.4	1.1
	MOL003166	Swertiamarin	374.38	-2.57	21.9	-1.29	-1.55
	MOL003527	Tyranton	116.18	-0.08	58.34	0.49	0.37
	MOL003546	Aristolone	218.37	3.19	45.31	1.29	1.54
	MOL003547	Azaron	208.28	2.74	38.39	1.42	1.18
	MOL000357	Sitogluside	576.95	6.34	20.63	-0.14	-0.93

	MOL000358	beta-sitosterol	414.79	8.08	36.91	1.32	0.99
	MOL003788	Cinnamein	238.3	3.74	78.8	1.37	1.02
	MOL003789	Coumaran	120.16	1.89	50.9	1.69	1.93
	MOL003877	Fuseloel	88.17	1.22	65.67	1	1.17
	MOL000041	PHA	165.21	0.96	41.62	0.36	0.22
	MOL000431	coumarin	146.15	1.9	29.17	1.2	1.3
	MOL000449	Stigmasterol	412.77	7.64	43.83	1.44	1
	MOL004582	Methyl naphthalene	142.21	3.23	39.01	1.9	1.91
	MOL004741	(7aR)-4,4,7a-trimethyl-6,7-dihydro-5H-benzofuran-2-one	180.27	2.48	40.48	1.2	1.39
	MOL000475	anethole	148.22	2.77	32.49	1.75	1.81
	MOL004784	Stenol	270.56	7.36	12.66	1.33	0.92
	MOL005022	1,8-DIMETHYLNAPHTHALENE	156.24	3.71	42.8	1.91	1.93
	MOL000511	ursolic acid	456.78	6.47	16.77	0.67	0.07
	MOL001996	Betulonic acid	454.76	6.48	16.83	0.65	0.14
	MOL005449	h-Met-h	149.24	-0.27	70.87	0.06	-0.17
	MOL005467	Epicatechin gallate	442.4	3.16	17.89	-0.55	-1.33
	MOL005472	1,2-Benzenedicarboxylicacid, mono(2-ethyl) hexylester	278.38	4.31	55.17	0.57	0.35
	MOL005474	1-Acetyl-4,6,8-trimethylazulene	212.31	3.94	27.02	1.48	1.4
	MOL005478	11,14-Octadecadienoic acid, methyl ester	294.53	6.64	41.93	1.5	1.25
	MOL005481	2,6,10,14,18-pentamethylicosa-2,6,10,14,18-pentaene	342.67	9.51	33.4	1.94	1.99
	MOL005482	FOA	112.09	0.83	35.66	0.63	0.74
	MOL005483	2-Methylnaphthalene	142.21	3.23	33.69	1.9	1.9
	MOL005484	GRO	150.19	1.93	52.16	0.89	1.06
	MOL005485	3-dibenzofuransulfonic acid	248.27	2.3	74.42	0.61	0.06
	MOL005486	3,4-Dehydrolycopen-16-al	548.92	11.48	46.64	2	0.6
	MOL005487	5-Carboxyresorcinol	154.13	0.9	69.34	0.01	-0.41
	MOL005488	3,5-Di-t-butyl-4-hydroxybenzaldehyde	234.37	4.12	27.19	1.21	1.19
	MOL005489	3,6-Digalloylglucose	484.4	-0.03	31.42	-1.95	-3.05
	MOL005490	4-(4-ethylphenyl)benzoic acid	226.29	3.9	42.06	1.14	0.74
	MOL005491	4-Ethyl-o-xylene	134.24	3.75	39.27	1.86	2.01
	MOL005492	4-Methoxy-1,2-benzodioxole	152.16	1.97	57.08	1.1	0.96
	MOL005493	6-Isopropyl-1,4-dimethylnaphthalene	198.33	4.91	33.79	1.91	1.91
	MOL005494	6-Tetradecanesulfonic acid,butyl ester	334.63	6.83	21.36	1.29	0.89
	MOL005495	6-ethyl-2,5-dihydroxy-1,4-na	218.22	1.87	20.58	0.39	-0.07

		phthoquinone					
	MOL005496	7,8-Dehydropenstemoside	404.41	-2.84	3.41	-1.75	-2.05
	MOL005497	7,8-Dehydropenstemoside_qt	242.25	-1.1	108.33	-0.33	-0.36
	MOL005498	7-Hydroxycadalene	214.33	4.64	29.3	1.67	1.57
	MOL005500	linolenate	278.48	5.95	45.01	1.22	0.95
	MOL005502	Butoxysuccinic Acid	190.22	0.78	37.55	-0.07	-0.24
	MOL005503	Cornudentanone	378.56	4.97	39.66	0.47	0.09
	MOL005505	Cornusiin B	1,086.77	3.62	17.84	-3.5	-4.83
	MOL005508	Glucosol	472.78	5.5	15.86	-0.08	-0.75
	MOL005510	D-1-O-Methyl mucoinositol	194.21	-2.66	25.11	-1.4	-3.64
	MOL005511	D-delta-tocopherol	402.73	9.44	16.36	1.6	1.45
	MOL005512	Dehydromorroniaglycone	226.25	0.05	58.78	0.25	0.17
	MOL005513	DBF	168.2	3.33	37.28	1.78	1.62
	MOL005514	Ethyl,alpha-hydroxymyristate	272.48	5.37	18.71	0.78	0.4
	MOL005515	Eugenone	252.29	1.29	18.7	0.43	0.24
	MOL005516	Fluoren	166.23	3.49	18.94	1.92	1.8
	MOL005522	LEN	87.19	0.93	62.94	1.01	1.02
	MOL005530	Hydroxygenkwanin	300.28	2.32	36.47	0.52	-0.44
	MOL005531	Telocinobufagin	402.58	2.11	69.99	-0.12	-0.85
	MOL008457	Tetrahydroalstonine	352.47	2.66	32.42	0.9	0.33
	MOL005534	trans-Verbenol	152.26	1.84	52.01	1.17	1.39
	MOL005535	Vitamin B1.mol2	266.41	-1.21	49.33	-1.43	-2.42
	MOL005536	Z,Z-10,12-Hexadecadien-1-ol acetate	280.5	5.94	44.09	1.43	1.26
	MOL005537	Z-5-Nonadecene	266.57	8.6	18.81	1.9	2.07
	MOL005538	Linolenyl alcohol	264.5	6.02	42.79	1.39	0.97
	MOL005539	Acenaphthylene	152.2	2.9	28.55	1.87	1.88
	MOL005540	alpha-Corocalene	200.35	5.03	13.97	1.9	1.91
	MOL005542	camptothin A	1,571.17	3.94	6.27	-5.36	-6.82
	MOL005543	camptothin B	1,086.77	3.45	3.01	-3.56	-4.62
	MOL005545	cornin_qt	226.25	-0.52	25.1	-0.2	-0.37
	MOL005546	cornuside	542.54	-0.71	2.61	-2.09	-2.98
	MOL005549	Dehydromevalonic lactone	112.14	1.16	53.46	1.12	1.39
	MOL005550	Vanirom	166.19	1.65	28.07	0.87	0.75
	MOL005552	gemin D	634.49	0.73	68.83	-2.17	-2.71
	MOL005553	2-Monopalmitin	330.57	5.57	26.74	0.39	-0.12
	MOL005554	i-Butanol	74.14	0.83	28.26	1.03	1.26
	MOL005555	isoterchebin	954.7	1.35	3.01	-3.87	-4.47
	MOL005556	1-tert-Butyl-7-methoxynaphthalene	214.33	4.12	27.46	1.74	1.61
	MOL005557	lanosta-8,24-dien-3-ol,3-acetate	468.84	8.5	44.3	1.45	1.31
	MOL005559	Maslinic acid	472.78	5.46	15.54	0.1	-0.55

	MOL005561	Anisylacetone	178.25	1.9	19.69	1.21	1.08
	MOL005562	phosphonofluoridic acid,(l-methylethyl)-,cyclohexyl ester	208.24	2.33	76	1.06	0.99
	MOL005563	(-)-Pulegone	152.26	2.75	51.23	1.4	1.65
	MOL005567	vitamin a	286.5	5.32	19.53	1.38	0.86
	MOL000057	DIBP	278.38	3.92	49.63	0.85	0.68
	MOL000570	Nonox D	219.3	4.29	39.82	1.88	1.61
	MOL000635	vanillin	152.16	1.31	52	0.68	0.41
	MOL000749	Linoleic	280.5	6.39	41.9	1.23	0.81
	MOL000971	Ethylpalmitate	284.54	6.97	18.99	1.41	1.15
Poria cocos	MOL000274	3β-hydroxylanosta-7,9(11),24-trien-21-oic acid	454.76	6.58	24.92	0.53	-0.24
	MOL000275	trametenolic acid	456.78	7.03	38.71	0.52	-0.14
	MOL000276	7,9(11)-dehydropachymic acid	526.83	6.1	35.11	0.03	-0.87
	MOL000277	tumulosic acid	486.81	6.16	15.95	0.02	-0.51
	MOL000279	Cerevisterol	430.74	5.15	37.96	0.28	-0.39
	MOL000281	Dimethyl L-malate	162.16	-0.45	8.59	-0.05	-0.61
	MOL000282	ergosta-7,22E-dien-3beta-ol	398.74	7.18	43.51	1.32	0.91
	MOL000283	Ergosterol peroxide	430.74	7.17	40.36	0.84	0.34
	MOL000287	3beta-Hydroxy-24-methylene-8-lanostene-21-oic acid	470.81	7.33	38.7	0.61	-0.04
	MOL000288	pachyman	500.56	-4.27	0.45	-2.36	-3.58
	MOL000289	pachymic acid	528.85	6.54	33.63	0.1	-0.57
	MOL000290	Poricoic acid A	498.77	5.94	30.61	-0.14	-0.93
	MOL000291	Poricoic acid B	484.74	5.64	30.52	-0.08	-0.87
	MOL000292	poricoic acid C	482.77	7.11	38.15	0.32	-0.41
	MOL000293	poricoic acid D	514.77	4.73	22.38	-0.62	-1.29
	MOL000296	hederagenin	414.79	8.08	36.91	1.32	0.96
	MOL000297	Tumulosic acid	486.81	6.16	29.88	0.13	-0.47
	MOL000299	Trimethyl citrate	234.23	-0.64	67.61	-0.06	-0.33
	MOL000300	dehydroeburicoic acid	453.75	6.35	44.17	0.38	-0.16
	MOL000301	2-lauroleic acid	198.34	4.52	31.42	1.03	1.08
	MOL000302	Undekansaeure	186.33	4.09	30.14	0.98	0.94
	MOL000303	caprylic acid	144.24	2.72	16.4	0.9	1.02
	MOL000305	lauric acid	200.36	4.54	23.59	1.02	1.1
	MOL000069	palmitic acid	256.48	6.37	19.3	1.09	1
Alisma orientale	MOL000359	sitosterol	414.79	8.08	36.91	1.32	0.87
	MOL000472	emodin	270.25	2.49	24.4	0.22	-0.66
	MOL000748	HMF	126.12	0.67	45.07	0.05	-0.27
	MOL000823	1h-indole-3-carboxylic,acid	161.17	1.73	25.83	0.79	0.61
	MOL000828	Alisol A	490.8	3.88	18.28	-0.48	-0.91

	MOL000829	Alisol A monoacetate	532.84	4.26	24.21	-0.13	-0.61
	MOL000830	Alisol B	472.78	4.64	34.47	0.04	-0.54
	MOL000831	Alisol B monoacetate	514.82	5.02	35.58	0.46	-0.18
	MOL000833	alisol,f	528.85	3.7	14.2	-0.01	-0.69
	MOL000837	lismol	220.39	3.58	49.43	1.27	1.27
	MOL000840	orientalol,f	236.39	2.76	73.8	1.15	1.21
	MOL000844	Sulfoorientalol A	302.48	2.14	28.14	0.27	-0.19
	MOL000846	Sulfoorientalol C	300.46	1.94	41.57	0.06	-0.22
	MOL000847	Sulfoorientalol D	318.48	1.04	18.72	0.38	0.21
	MOL000849	16β-methoxyalisol B monoacetate	544.85	4.33	32.43	0.07	-0.6
	MOL000850	alisol A	490.8	3.88	18.28	-0.2	-0.83
	MOL000851	alisol A(B,C)monoacetate	532.84	4.26	24.21	-0.28	-1.11
	MOL000852	16β-hydroxyalisol B monoacetate	530.82	3.92	29.94	-0.25	-0.97
	MOL000853	alisol B	444.72	3.76	36.76	0.07	-0.67
	MOL000854	alisol C	486.76	3.5	32.7	-0.34	-0.97
	MOL000855	alisol B monoacetate	515.78	3.32	29.67	-0.51	-0.78
	MOL000856	alisol C monoacetate	514.77	3.67	33.06	-0.18	-0.73
	MOL000857	NCA	122.14	-0.32	71.13	0.44	0.2
	MOL000858	Glycerol palmitate	330.57	5.57	26.66	0.17	-0.5
	MOL002464	1-Monolinolein	354.59	5.59	37.18	0.32	-0.32
	MOL000860	stearic acid	284.54	7.28	17.83	1.15	1.22
	MOL000861	Healip	326.68	9.18	11.65	1.38	0.8
Rehmannia glutinosa	MOL000012	Arachic acid	312.6	8.19	16.66	1.18	1.09
	MOL001501	Daturic acid	270.51	6.82	18.51	1.12	0.95
	MOL001709	methyl (2E,4E)-hexadeca-2,4-dienoate	266.47	6.15	41.57	1.37	1.23
	MOL001739	zoomaric acid	254.46	5.92	35.78	1.18	0.88
	MOL002819	catalpol	362.37	-3.77	5.07	-1.72	-2.33
	MOL000360	FER	194.2	1.62	39.56	0.47	-0.03
	MOL003689	aeginetic acid	268.39	2.24	48.31	0.13	-0.23
	MOL003692	Ajugoside_qt	212.27	0.35	81.68	0.24	-0.13
	MOL003695	Cistanoside A	800.84	-1.11	3.4	-2.72	-4.08
	MOL003708	jioglutin D	246.29	-1.42	39.02	-0.22	-0.81
	MOL003709	jioglutin E	232.31	-0.72	81.9	-0.04	-0.59
	MOL003710	jioglutolide	186.23	-0.93	86.95	-0.15	-0.55
	MOL003713	Jioglutoside B_qt	210.25	0.64	89.22	0.29	0.04
	MOL003717	METHYL PALMITOLEATE	268.49	6.17	34.61	1.38	1.32
	MOL003719	methyl-2,6,10-trimethyltridecanoate	269.5	5.7	24.86	0.85	0.74
	MOL003721	Rehmaglutin B	236.67	-1.03	64.62	-0.55	-1.11

	MOL003725	Rehmaionoside C	388.51	-0.44	12.89	-1.18	-1.42
	MOL003730	Rehmannioside A	524.53	-5.51	25.95	-3	-4.43
	MOL003731	Rehmaglutin A	202.23	-1.63	29.7	-0.82	-1.25
	MOL003732	Rehmaglutin D	220.67	-0.43	57.03	-0.17	-0.61
	MOL003733	6-O-vanilloylajugol	484.5	-1.14	23.44	-1.26	-1.95
	MOL003734	echinacoside	786.81	-1.36	3.14	-2.91	-4.05
	MOL003735	aucubin	346.37	-3.1	4.17	-1.83	-2.9
	MOL000399	Docosanoate	340.66	9.11	15.69	1.21	0.91
	MOL000449	Stigmasterol	412.77	7.64	43.83	1.44	1
Dioscorea opposita	MOL000136	Dioscoreside C	1,061.36	-1.25	10.32	-3.13	-3.87
	MOL001559	piperlonguminine	273.36	2.93	30.71	0.95	0.27
	MOL001618	Pellitorin	223.4	3.96	23.81	1.36	1.1
	MOL001736	(-)-taxifolin	304.27	1.49	60.51	-0.24	-1.02
	MOL002442	Cholesteryl ferulate	562.91	9.6	22.43	1.09	0.45
	MOL000309	denudatin,a	340.4	2.61	6.93	0.71	0.21
	MOL000310	Denudatin B	356.45	2.8	61.47	0.9	0.35
	MOL000322	Kadsurenone	356.45	2.8	54.72	0.82	0.52
	MOL000388	gamma-aminobutyric acid	103.14	-0.62	24.09	-0.26	-0.57
	MOL000041	PHA	165.21	0.96	41.62	0.36	0.22
	MOL000042	LPG	89.11	-0.6	87.69	-0.34	-0.77
	MOL004668	GLB	180.18	-2.51	47.71	-1.89	-4.41
	MOL005367	GUP	180.18	-2.51	43.04	-1.82	-4.46
	MOL000054	L-	174.24	-1.11	47.64	-0.49	-1.04
	MOL005428	(24S)-belta-Methyl cholestanol	402.78	7.88	25.81	1.29	0.98
	MOL005429	hancinol	372.5	2.46	64.01	0.53	0.17
	MOL005430	hancinone C	400.51	3.34	59.05	0.74	0.06
	MOL005431	Crotepoxide	362.36	0.59	27.02	0.08	-0.1
	MOL005432	ABK	264.35	2	63.67	0.13	-0.2
	MOL005438	campesterol	400.76	7.63	37.58	1.34	0.95
	MOL005439	Ostreasterol	398.74	7.43	13.62	1.31	0.88
	MOL005440	Isofucosterol	412.77	7.83	43.78	1.36	0.97
	MOL005441	BGC	180.18	-2.51	48.09	-2.02	-4.68
	MOL005442	LDP	153.2	0.72	74.4	0.3	-0.21
	MOL005443	Batatasin I	284.33	3.33	23.7	1.06	0.52
	MOL000298	ergosterol	396.72	6.93	14.29	1.47	1.04
	MOL005446	Batatasin IV	244.31	3.71	82.76	1.09	0.7
	MOL005447	L-Xyl	150.15	-2.17	40.44	-1.26	-1.78
	MOL005448	Leucinum	131.2	0.63	72.92	-0.05	-0.37
	MOL005449	h-Met-h	149.24	-0.27	70.87	0.06	-0.17
	MOL005450	cholestanol	388.75	7.63	25.63	1.3	0.99
	MOL005451	holest-7-enol	386.73	7.38	11.81	1.32	1
	MOL000449	Stigmasterol	412.77	7.64	43.83	1.44	1

	MOL005454	(S)-Allantoin	158.14	-1.76	106.68	-0.82	-0.98
	MOL005455	(-)-ABA	264.35	2	31.79	0.06	-0.26
	MOL005456	Deltoside	1,063.33	-1.93	10.07	-3.67	-4.43
	MOL005457	Deltoside_qt	430.69	4.17	10.36	0.27	-0.28
	MOL005458	Dioscoreside C_qt	444.72	3.96	36.38	0.39	-0.44
	MOL000546	diosgenin	414.69	4.63	80.88	0.82	0.27
	MOL005460	ophipogonin D_qt	414.69	4.63	15.84	0.8	0.21
	MOL005461	Doradexanthin	584.96	8.73	38.16	0.52	-1.19
	MOL005462	Methylcimicifugoside	688.94	1.97	46.31	-0.58	-1.59
	MOL005463	Methylcimicifugoside_qt	556.81	3.21	31.69	0.21	-0.41
